# Exploring Head and Neck Paraganglioma: A Case Report

**DOI:** 10.7759/cureus.55720

**Published:** 2024-03-07

**Authors:** Soumiya Samba, Ahmed Bensghier, Souad Margoum, Soufiane Berhili, Mohamed Moukhlissi, Loubna Mezouar

**Affiliations:** 1 Department of Radiation Oncology, Mohammed VI University Hospital, Oujda, MAR; 2 Faculty of Medicine and Pharmacy, Mohammed First University, Oujda, MAR

**Keywords:** pheochromocytoma, autonomic nerve system, cancer, head and neck, paraganglioma

## Abstract

Paragangliomas (PGLs) are tumors that are rarely malignant; the majority of them are benign. Similar to pheochromocytoma, they develop from the autonomic nerve system. This system originates from neural crest cells and can undergo neoplastic transformation. PGLs can arise either inside or outside the adrenal glands. Head and neck PGLs are very scarce. The primary locations where this tumor commonly originates within this region are the carotid body, jugular bulb, and vagal body. Hence, in our case report, we attempt to highlight the uncommon presentation of this disease in a 46-year-old female, who initially presented with hypertension and persistent dysphonia. The patient underwent successful external radiotherapy. This case report aims to raise awareness of the characteristics of these rare malignancies.

## Introduction

Head and neck paragangliomas (PGLs) demonstrate a notable rarity, with an incidence rate of 0.0012% [1]. Commonly, these tumors manifest symptoms that may be erroneously attributed to other benign or malignant neoplasms [1]. PGLs are frequently misdiagnosed as glomus tumors, primarily due to their shared characteristic of high vascularity, despite having different immunological and histological features [2]. The most commonly encountered types include the carotid (carotid body), jugular (jugular bulb), and vagal (vagal nerve) PGL [3].

The clinical presentation of PGLs may vary based on factors such as the tumor's anatomical location, its functional activity, and other concurrent factors. Infrequently, patients may show no symptoms at all. Early diagnosis of these tumors is essential to prevent numerous complications, and this can be challenging due to its various clinical presentation. Hence, early diagnosis necessitates a high level of suspicion.

## Case presentation

A 46-year-old female, with a past medical history of non-insulin-dependent type II diabetes mellitus being treated with metformin 750 mg twice daily for the past 10 years, presented with a history of hypertension, vertigo, and chronic dysphonia over the past year and a half. The symptomatology worsened with the emergence of intermittent episodes of facial paralysis, characterized by anarchic frequency, occurring sporadically, ranging from once a day to once every few weeks.

**Figure 1:**
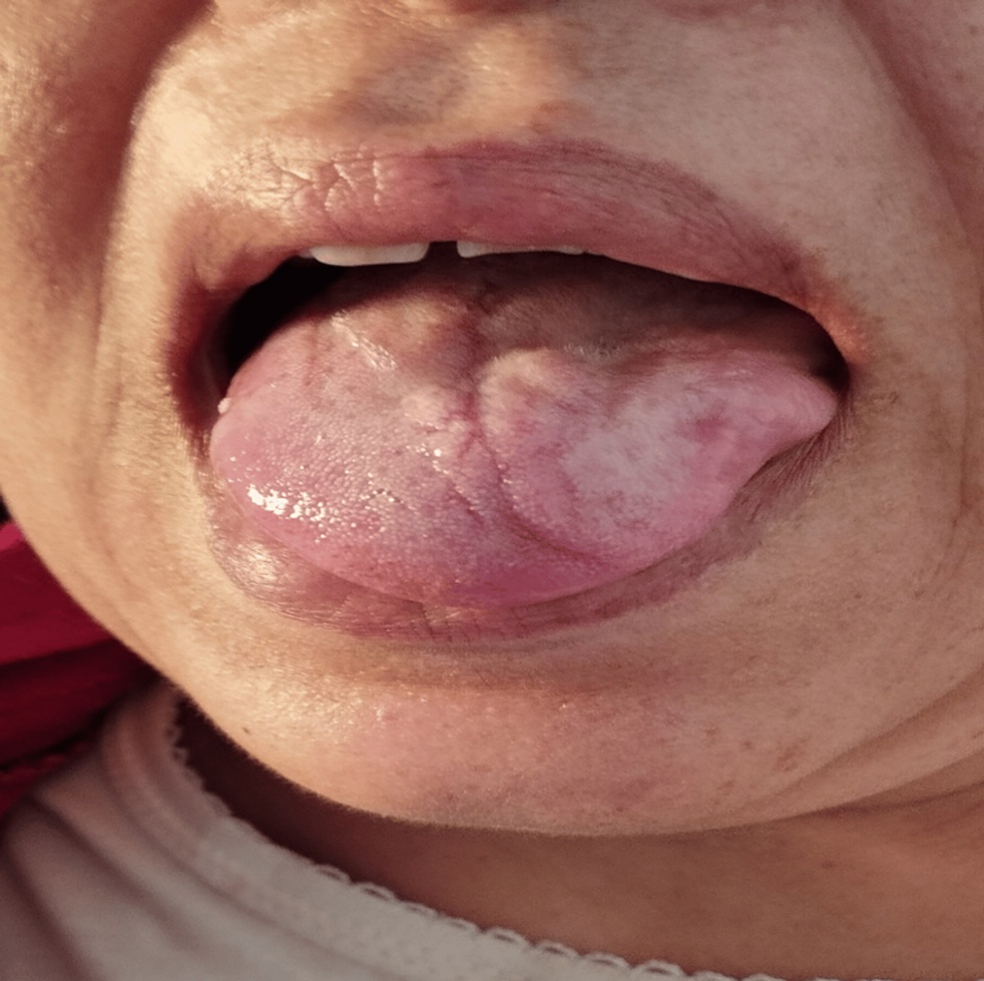
Physical examination showing a lingual deviation

**Figure 2:**
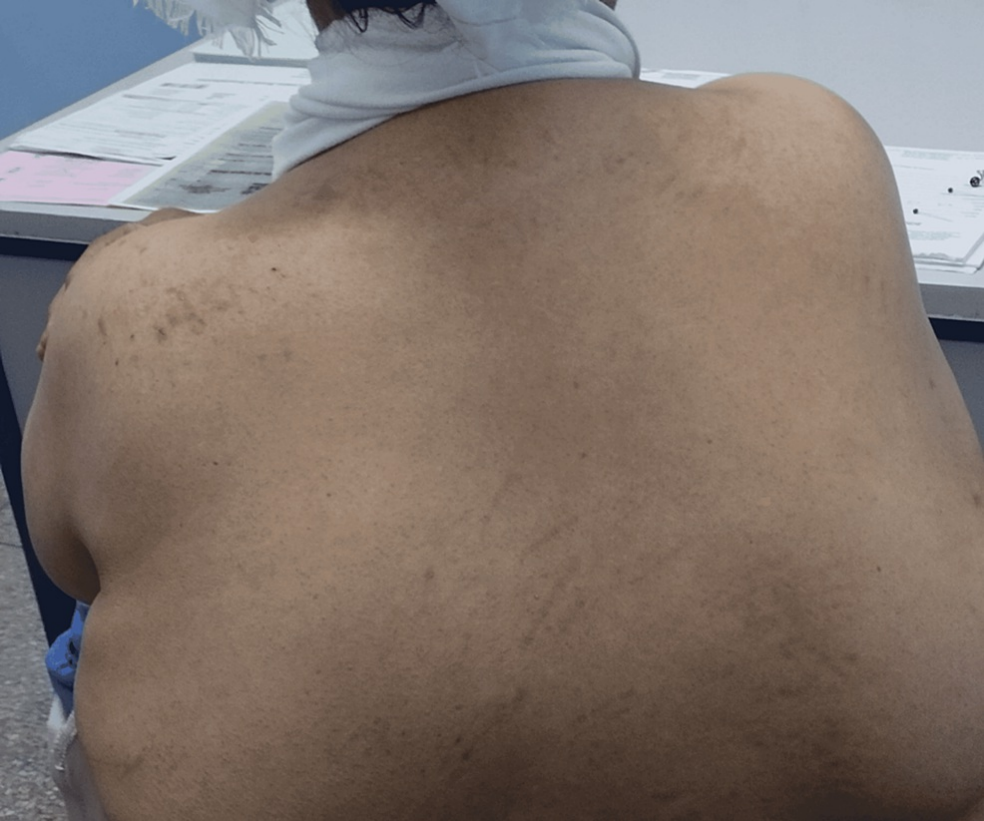
Clinical examination showing a falling left shoulder with amyotrophy of the trapezius muscle

**Figure 3:**
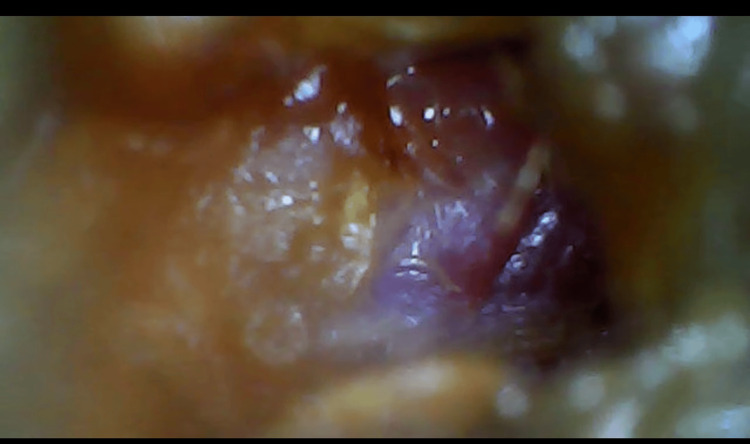
Otoscopic examination indicated the presence of a cyan mushroom-like growth in the tympanic membrane.

On examination, the patient was conscious with normal vital signs, well-oriented in time and space, and demonstrated hemodynamic and respiratory stability. On physical examination, we noted a tongue deviation to the left (Figure 1) and a drooping left shoulder with amyotrophy of the trapezius muscle (Figure 2), while the otoscopic examination of the left ear showed a blue bump over the tympanic membrane (Figure 3). The rest of the examination revealed no abnormalities.

**Figure 4:**
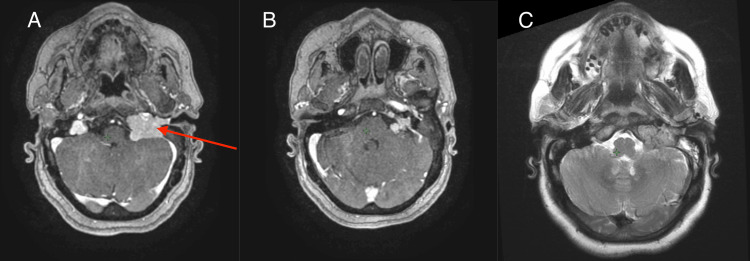
Cerebral MRI showing a left tympano-jugular tumor process (red arrow) measuring 39 x 29 mm responsible for osteolysis of the jugular foramen, with lobulated contours, containing flow voids on the T2 sequence with intense, homogenenous enhancement after contrast, evoking a paraganglioma A: T1 injected; B: T1 non-injected; C: T2

A cerebral MRI was performed (Figure 4) and showed a right tympano-jugular tumor process measuring 39 x 29 mm, responsible for osteolysis of the jugular foramen. It displayed lobulated contours and contained flow voids on the T2 sequence with intense, homogeneous enhancement after contrast, evoking a PGL.

**Figure 5:**
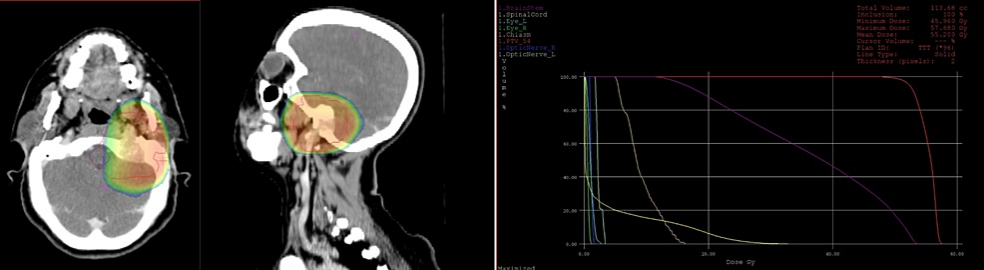
Radiotherapy was administered following the recommended doses for the brainstem

The staging evaluation revealed no abnormalities and urinary catecholamines were also negative. The neurosurgery team declined to perform the biopsy due to the delicate location of the tumor. However, the radiology team confirmed that the MRI displayed the typical aspect of a PGL. Therefore, the decision was to treat the patient with three-dimensional conformal external radiotherapy only. The total dose delivered was 54 Gy, divided into 27 fractions of 2 Gy each. This approach was favored due to the inoperability of the tumoral process, as well as the presence of multiple cranial nerve paralysis induced by the extension of osteolysis. Radiotherapy was administered while adhering to the recommended doses for the brainstem (Figure 5). One month after the last dose, the patient reported no complaints and showed a favorable clinical evolution, with regression of the previously reported symptoms.

## Discussion

PGLs are a very uncommon type of highly vascular neuroendocrine tumors with the potential to produce and release catecholamines (dopamine, norepinephrine, and epinephrine) [4]. They exhibit a 25% occurrence rate in individuals aged 25 years or younger [5]. Genetic mutation, inherited in a dominant autosomal way, affects both men and women equally [6-8]. The majority of PGL show genomic alterations mainly in the DNA of genes coding for succinate dehydrogenase (SDH), an enzyme located in complex II of the mitochondrial respiratory chain [5].

Diagnosing PGLs may be challenging as they can manifest in various ways and have three essential components: clinical, biological, and radiological [6]. The primary cause of this condition is the secretion of catecholamines, particularly noradrenaline. In terms of the pathobiology of this process, multiple systemic effects may occur, giving rise to various symptoms. These include persistent constipation, chest pain, and tachycardia in the cardiovascular system, mydriasis and cranial nerve paralysis in the neurologic system, increased urination and thirst in the endocrine system, and psychological manifestations such as panic attacks and agitation, along with musculoskeletal fatigue [6].

A secretory PGL, meaning it secretes catecholamines, is often diagnosed by detecting urine and/or plasma-fractionated metanephrines and catecholamines. Biochemical tests are essential for all cases of PGL, regardless of clinical manifestations. Similar to other neuroendocrine tumors, higher levels of antisera to neuron-specific enolase (NSE), chromogranin A, or vimentin in the circulation may be useful in identifying these tumors from non-neuroendocrine malignancies [7].

Imaging by MRI is the third important element of the diagnosis of PGL and complements the clinical examination. Regularly used techniques include ultrasound imaging, triple-phase helical CT, gadolinium-diethylenetriaminepentaacetic acid perfusion MRI, angiography, and radioisotope imaging using metaiodobenzylguanidine (MIBG) and fluorodeoxyglucose (FDG)-positron emission tomography (PET) [8]. PGLs are often presented as iso or hypointense on T1-weighted MRI scans and show high signal intensity on T2-weighted imaging, particularly when fat suppression is used. While the sensitivity of this result is notably high, its specificity is lacking [9].

The extensive blood supply associated with PGL often complicates their surgical removal, leading to increased risks of excessive bleeding during the procedure. Preoperative embolization has shown a significant ability to reduce blood loss during surgery, principally in jugulo-tympanic PGL. For radiotherapy and radiosurgery, both are recommended and can control long-term tumor growth and reduce catecholamine excretion in functional tumors [10].

However, given the restricted duration of observation following stereotactic radiosurgery, conclusive interpretations cannot be made [11]. The choice of treatment depends on factors such as the size and location of the tumor, the expected side effects of available treatment options, the patient's age and well-being, and the preferences of both the patient and the clinician. Both surgical intervention and radiation provide a good potential for achieving local control. The main difference between these modalities of treatment is the level of associated morbidity, which is determined by the tumor's location and volume. Thus, the possibility of achieving local control after radiation therapy remains independent of the tumor's size or location [12].

Observation is an alternative treatment option that might be considered for asymptomatic patients with a poor prognosis. Nevertheless, clinicians choosing this strategy must be cognizant that cranial nerve deficits induced by tumor growth are frequently irreversible. This consideration should be carefully weighed against the comparatively minor morbidity associated with radiotherapy [13].

PGLs are uncommon neuroendocrine malignancies presenting significant risks and carrying a substantial probability of causing mortality or disability [14]. These tumors are differentiated by their tendency to emerge in various locations and their aggressive nature. Approximately 10% of PGLs are malignant, and the likelihood of survival beyond five years post the diagnosis is less than 50% [15].

## Conclusions

This case report highlights the uncommon presentation of head and neck PGLs, pointing out the diagnostic challenges associated with their rarity. Effective treatment with external radiation demonstrates the value of early identification. Clinicians should be vigilant of these tumors since they frequently disguise as other neoplasms, particularly glomus tumors. This contribution aims to promote understanding and timely diagnosis of head and neck PGLs, leading to better patient outcomes due to increased medical professional awareness and knowledge.
